# Early minimally invasive image-guided eNdoscopic evacuation of iNTracerebral hemorrhage: a phase II pilot trial

**DOI:** 10.3389/fneur.2024.1484255

**Published:** 2024-11-19

**Authors:** Tim Jonas Hallenberger, Urs Fischer, Nilabh Ghosh, Jens Kuhle, Raphael Guzman, Leo Hermann Bonati, Jehuda Soleman

**Affiliations:** ^1^Department of Neurosurgery, University Hospital Basel, Basel, Switzerland; ^2^Faculty of Medicine, University of Basel, Basel, Switzerland; ^3^Department of Neurology, University Hospital Basel, Basel, Switzerland; ^4^Department of Biomedicine, University of Basel, Basel, Switzerland; ^5^Rehabilitation Center Rheinfelden, Rheinfelden, Switzerland

**Keywords:** endoscopic surgery, intracerebral hemorrhage, functional outcome, pilot trial, neurosurgery

## Abstract

**Background:**

Whether minimally invasive endoscopic surgery (ES) improves survival and functional outcome in people with spontaneous supratentorial intracerebral hemorrhage (SSICH) is unknown.

**Methods:**

This is a single-center pilot study performed between July 2021 to January 2023. Any supratentorial hematoma with a volume between 20 mL and 100 mL was endoscopically evacuated within 24 h after bleeding onset. Participants were followed-up for 6 months, assessing clinical and radiological outcomes. The primary feasibility outcome was satisfactory hematoma removal (<15 mL residual volume on the first postinterventional CT study) and the primary efficacy outcome was reaching a modified Rankin Scale 0–3 (mRS) at 6 months. Secondary outcomes were mortality and morbidity rates.

**Results:**

Ten participants (median age 72.5 years [IQR 67–81], 70% male, median baseline hematoma volume 34.1 [IQR 25.5–58.0]) were included. Satisfactory hematoma evacuation was achieved in 70% (7/10) with a median evacuation percentage of 69.5% [IQR 45.3–93.9%]. The median duration of surgery was 91 min [IQR 73–111]. Favorable outcome at 6 months was observed in 60% of the participants and improved from within 24 h before the intervention to the last follow-up (6 months). Five participants (50%) experienced a total of six complications, two recurrent bleedings, three pneumonias and one epilepsy. Mortality rate was 30%, while one participant died from pneumonia, one from a recurrent bleeding, and one participant due to a glioblastoma.

**Conclusion:**

ES appears to be feasible, with satisfactory hematoma removal being achieved in the majority of participants. Based on the descriptive results of this pilot trial, a national multicenter RCT comparing ES to best medical treatment is currently ongoing

**Clinical trial registration:**

https://clinicaltrials.gov/, identifier NCT05681988.

## 1 Introduction

Intracerebral hemorrhage (ICH) is one of the most devastating forms of stroke, affecting around 3.41 million people annually (1, 2). Mortality rates reach up to 40% while the burden of disease for survivors remains catastrophic (3, 4). In SSICH, the hematoma volume directly contributes to poorer outcomes, due to its mass effect and toxic break down products causing further secondary damage, previous treatment trials aimed to prevent hematoma growth or reduce hematoma volume (5–13). As opposed to infratentorial ICH, where surgical treatment seems superior, in SSICH previous surgical trials failed to reduce mortality or improve functional outcome compared to best medical treatment (BMT) alone (5, 9, 10, 12–14). Therefore, BMT remains the mainstay of SSICH treatment, which includes a care bundle approach of a combination of active blood pressure management, intensive care unit surveillance, prevention of further complications and early rehabilitation (13).

Early minimal invasive endoscopic hematoma evacuation (ES) has been introduced years ago and showed some promising results (14, 15). A recent systematic review and meta-analysis showed a reduction in mortality and improved functional outcome after ES evacuation of SSICH compared to BMT. However, the included studies were of low power and quality (16). Recently the ENRICH trial has been completed showing for the first time beneficial outcome using minimally invasive surgery in lobar hematomas with current trials being ongoing to confirm these results (17). Furthermore, uncertainty remains about the ideal time point of the evacuation of SSICH. MISTIE III suggested an improved functional outcome in people treated early (<48 h) compared to a later treatment start (>48 h), however confirmatory studies are lacking (12, 18, 19). Given the current lack of evidence, further investigation regarding early, minimally invasive endoscopic evacuation of SSICH is needed.

We herein report our phase II pilot trial, assessing feasibility and safety of early minimally invasive endoscopic surgery for SSICH.

## 2 Methods

In this single-center, phase II, prospective pilot trial we included people between 18 and 85 years of age, without disabilities prior to SSICH (mRS 0–1), with any supratentorial lobar or deep hematomas of a volume between 20 and 100 mL and a neurological deficit, assessed with the NIH Stroke Scale Score (NIHSS) of ≥8 points or a Glasgow Coma Scale (GCS) between 5 and 13 points (20). People with vascular malformations, tumor, or trauma as well as people with active bleeding or coagulation disorder (i.e., spot sign and an INR of >1.5) were excluded (full eligibility criteria can be found in Supplementary Table S1). A spot sign was defined as an extravasation of contrast agent as demonstrate by computed tomography angiography. Oral anticoagulation was not an exclusion criterion if the effects could be reversed before surgery (i.e., within 24 h after SSICH onset). Oral anticoagulants were reversed according to current guidelines for SSICH and our in-house recommendations using the recommended antidots if available or prothrombin complex concentrates (21). We planned to include at least 10 participants in this exploratory and feasibility study. No formal sample size calculation was done.

Consecutive enrolment took place at the emergency department of the University Hospital Basel from July 2021 until January 2023 with some delay in recruitment due to the COVID-19 pandemic. All participants included underwent ES for the removal of the SSICH within 24 h after symptom onset. Follow-up visits took place at 24 h and 3 days after surgery, as well as at discharge, 1 month, and 6 months (Supplementary Table S2). The surgical procedure has been previously reported (22). In brief, a trajectory with the shortest distance to the long axis of the hematoma is calculated in our navigational software (Brainlab Inc., Munich, Germany). After completing a burr-hole trepanation the endoscope is inserted through a transparent trocar and guided by navigation to the distal end of the hematoma cavity. Using constant irrigation and suction, the hematoma is evacuated from distal to proximal. After completing the hematoma evacuation a postoperative CT scan for quality control is completed (outside of the OR in a standard CT scanner) (22). In addition to surgery, BMT is applied in all participants according to institutional guidelines (13).

The primary feasibility outcome was a satisfactory reduction in hematoma volume to <15 mL residual volume directly after but not later than 24 h after surgery, measured by the volumetric function of our navigational software. For calculation of volumetric differences before and after surgery, high resolution CT scans were compared. This threshold was chosen in accordance with findings from previous studies, leading to increased odds of favorable outcome in affected patients (12, 23). The primary efficacy outcome was a modified Rankin Scale 0–3 (mRS) at 6 months. Secondary outcomes were mortality at 6 months, relative reduction of the hematoma volume (admission compared to the first postoperative CT scan) and neurological deficits measured by the NIHSS at 6 months. Additionally, consecutive blood sampling was performed to assess fluid biomarkers for brain damage which will be presented in a future report.

All data were collected prospectively. CT imaging studies were conducted upon admission, 24 h postoperative and 3 days after surgery (MRI). For screening and including participants into the study the AxBxC/2 method was used to measure hematoma volume, while all volumes were secondarily validated using the volumetric function of the Brainlab (Brainlab Inc., Munich, Germany) navigation station using the admission CT and the first CT after surgery (20).

Data were reported descriptively. No formal comparison of safety outcomes and functional outcomes was possible due to the lack of a comparator. Safety and functional outcome results were reported in context with contemporary literature of comparable trials (24–26). Only patients with completed follow-ups were included in the final analysis. Within group comparison (mRS admission vs. mRS 6-month follow-up; NIHSS admission vs. NIHSS 6-month follow-up and GCS admission vs. GCS 6-month follow-up) was done using Wilcoxon signed rank sum- or McNemars test. An alpha level of 5% was deemed significant. Statistical analysis was performed using SPSS (V 28, IBM, Armonk, NY, United States). This trial was approved by the local ethics committee (EKNZ 2021–00161) and conducted according to the Declaration of Helsinki (27). The trial was registered with the identifier NCT04805177 and reported in agreement with the Consort Statement and the extension for pilot and feasibility trials (28, 29). All participants were informed about the study and consented for participation. An anonymized dataset is available with the corresponding author upon reasonable request.

## 3 Results

**Figure 1:**
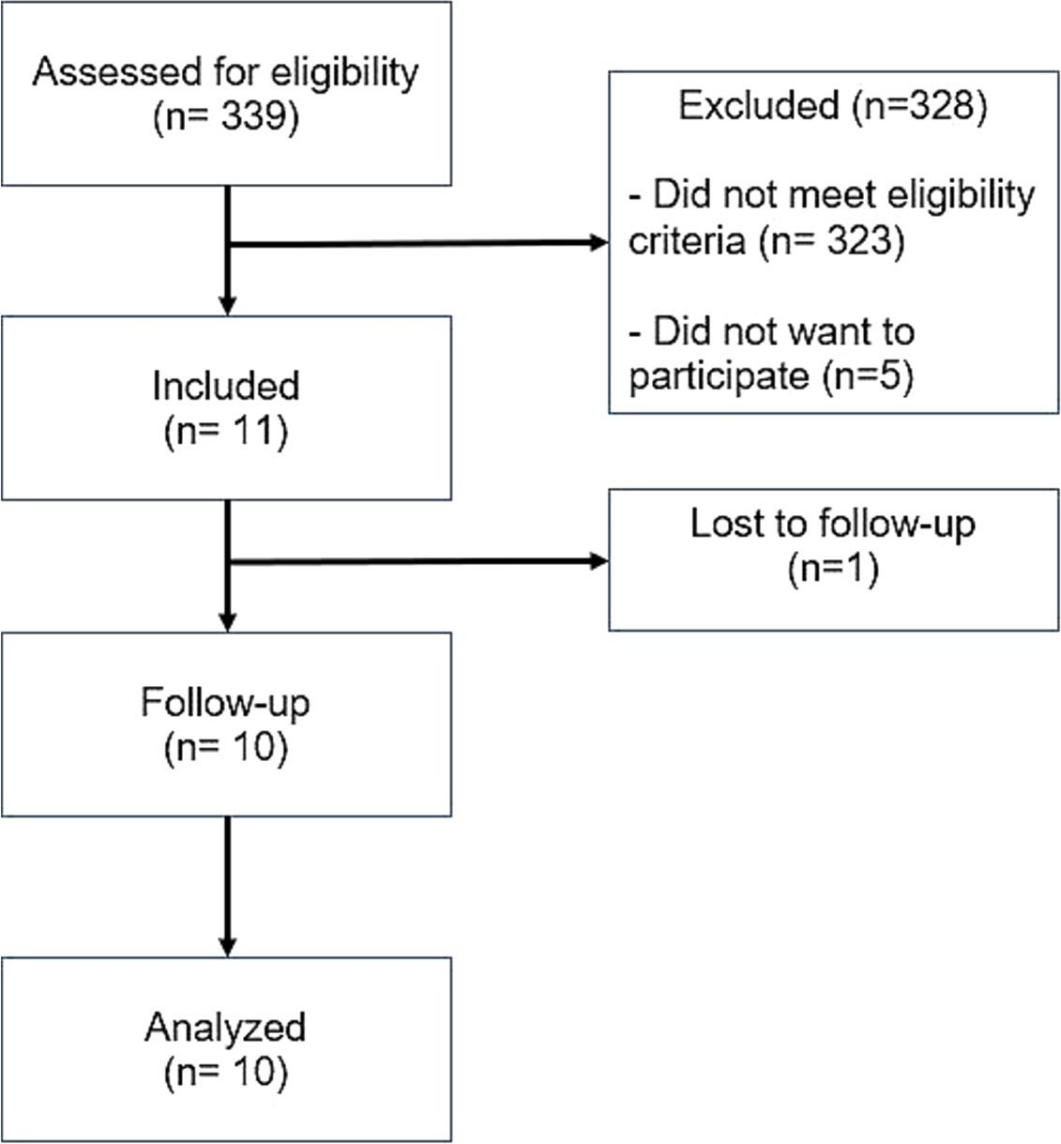
CONSORT flow chart for the study.

Between July 2021 and January 2023, 339 people with ICH of various etiologies, were screened. Sixteen participants were found eligible, and 11 were included. The most common reason for non-eligibility were the non-spontaneous etiology of the ICH (i.e., traumatic ICH) and inadequate hematoma volume (too large/too small). One participant was lost to follow-up (at visit 5/1 month after intervention) and 10 participants were included for the final analysis (Figure 1).

### 3.1 Baseline demographic data

The median age was 72.5 years (IQR 67–81) with a male predominance (*n* = 7, 70.0%). Deep-seated hematomas were more commonly observed (*n* = 6, 60%) with a median pre-interventional hematoma volume of 34.1 mL (IQR 25.5–58.0). Three participants (30%) suffered their ICH under oral anticoagulation. Five participants (50%) had a history of hypertension, while previous cerebrovascular ischemia, diabetes mellitus type II, epilepsy, and mild cognitive impairment were reported in one case each. Median baseline systolic blood pressure at admission was 150 (IQR 124–177) mmHg. Median baseline NIHSS score was 12 (IQR 10–18), median baseline GCS was 13 (IQR 11–14) and median mRS was 4 (IQR 4–5) points (Table 1).

**Table 1:** Baseline characteristics of the included patients.

Variable	N (%)/mean ± SD/median (IQR)
Age (years)	72.5 (67.0–81.0)
Male sex	7 (70)
Deep-seated hematoma	6 (60)
Superficial hematoma	4 (40)
Initial hematoma volume	34.1 (25.5–58.0)
Medical history	
Hypertension	5 (50)
Previous stroke	1 (10)
Diabetes mellitus type II	1 (10)
Epilepsy	1 (10)
Mild cognitive impairment	1 (10)
Systolic blood pressure at admission (mmHg)	150 (124–177)
Heart rate at admission (bpm)	80 (75–82)
Body temperature at admission (°C)	37.0 (36.0–37.0)
Medication at baseline	
Oral anticoagulants	3 (30)
BMI (kg/m^2^)	24.6 (22.5–27.2)
Baseline NIHSS	12 (10–18)
Baseline GCS	13 (11–14)
Baseline mRS	4 (4–5)

CVI, cerebrovascular insult; bpm, beats per minute; BMI, body mass index; NIHSS, National Institute of Health Stroke Scale; GCS, Glasgow Coma Scale; mRS, modified Rankin Scale.

### 3.2 Primary and secondary outcomes

Satisfactory hematoma evacuation was observed in seven participants (70%). We observed a trend toward improvement in the median evacuation rate from the first to the last participant (median evacuation rate of the first five participants: 62.5% (IQR 40–78%) and last five participants: 92.8% (IQR 47–96%), *p* = 0.251). We recorded six (60%) patients with favorable outcome (Tables 2, 3) with the primary efficacy outcome of a mRS 0–3 at 6 months. The location of the hematoma (deep vs. lobar) appeared to have no influence on satisfactory hematoma evacuation rate or functional outcome at 6 months. Satisfactory hematoma evacuation rate did not influence the rate of favorable outcome at 6 months.

**Table 2:** Development of neurological scores over time.

Variable/Timepoint	Admission (within 24 h after SSICH onset) (*n* = 10)	24 h after intervention (*n* = 10)	72 h after intervention (*n* = 10)	7d after intervention (*n* = 10)	1 m after intervention (*n* = 8)	6 m after intervention (*n* = 7)	p (Admission vs. 6 m after intervention)
mRS median (IQR)	4 (4–5)	4 (4–5)	4 (4–4)	4 (4–4)	3 (2–5)	2 (2–6)	0.097
mRS ≤3 n (%)	0 (0%)	0 (0%)	1 (10%)	2 (20%)	7 (70%)	6 (60%)	**0.031**
GCS median (IQR)	13 (11–14)	14 (9–14)	14 (13–14)	14 (13–15)	15 (15–15)	15 (15–15)	**0.024**
NIHSS median (IQR)	12 (10–18)	11.5 (8–17)	9 (8–13)	8.5 (6–15)	3 (1–4)	2 (0–6)	**0.028**

h, hours; d, days; m, months; NIHSS, National Institute of Health Stroke Scale; GCS, Glasgow Coma Scale; mRS, modified Rankin Scale. Bold values indicate statistically significant results.

**Table 3:** Surgical data and complications.

Variable	N (%)/mean ± SD/median (IQR)
Time to surgery (hours)	17 (12.5–22)
Duration of surgery (min)	91 (73–111)
Satisfactory hematoma evacuation (<15 mL)	7 (70%)
Hematoma reduction (mL)	29.84 (8.87–45.9)
Hematoma reduction (%)	69 (45–93)
Cases with serious adverse events	5 (50%)
Mortality	3 (30%)
Pneumonia	3 (30%)
Rebleeding	2 (20%)
Seizure	1 (10%)
ICU management	10 (100%)
Patients needing ventilation*	2 (20%)

*Extended mechanical ventilation after anaesthesia for surgery.

Three participants (30%) died. In two the cause of death may be related to the intervention or sequelae of the underlying disease, respectively, (pneumonia and recurrent hematoma with palliative treatment decision, both between hospital discharge and 1-month follow-up) and one participant died due to a GBM WHO grade IV not related to the intervention or the initial bleeding 6 months after intervention. Median change in hematoma volume was 29.8 (IQR 8.9–45.9) ml, corresponding to a median percentage of 69.51% (IQR 45.3–93.9%) hematoma reduction after surgery. Both, NIHSS and the GCS improved significantly at 6 months compared to baseline (*p* = 0.024 and *p* = 0.028, respectively).

Median duration of surgery was 91 (IQR 73–111) minutes (Table 3). Median time to surgery was 17 (IQR 12.5–22) hours after symptom onset while participants were treated in a postoperative ICU setting for the median time of 20 (IQR 15.5–87.0) hours.

### 3.3 Safety outcomes

A total of six adverse events were seen in five cases (50%); rebleeding (one patient within 72 h after intervention and one around 5 months after intervention. The latter patient presented with an underlying glioblastoma which led to rebleeding in the hematoma cavity) in two participants, pneumonia in three, and epilepsy in one participant (Table 3).

## 4 Discussion

The EMINENT-ICH pilot trial showed technical feasibility of early minimally invasive endoscopic hematoma evacuation as well as a good safety profile over time without being able to prove efficacy of the intervention by lack of a control group in this pilot study.

### 4.1 Feasibility and technique of endoscopic hematoma evacuation

This study primarily assessed the feasibility of ES, which is not routinely offered for SSICH removal. Therefore, and according to the IDEAL guidelines, a phase II pilot study is mandatory before conducting a large multicenter randomized trial (30). Due to our small sample size, we refrained from performing an assessment of a potential learning curve. Nevertheless, both junior and senior attendings showed a swift understanding of the technique and key learning points (workflow with the transparent trocar, planning the access and trajectory) could be rapidly implemented. Consequently, the median evacuation rate could be improved from the first to the last participant (median evacuation rate of the first five participants: 62.5% (IQR 40–78%) and last five participants: 92.8% (IQR 47–96%), *p* = 0.251), similar to reports from previous studies (26, 31). With a median evacuation rate of 69.5% and a proportion of satisfactory hematoma evacuations of 70%, we are well within the technical efficacy reported in previous studies ranging from 54 to 97% (12, 25, 31–34). Our technique is based on the SCUBA technique described by Kellner et al. (22, 35). However, our technique differs from other trials using either the Apollo/Artemis device (DIST, MIND) or the NICO Myriad system (ENRICH) (22, 26, 35–37). We use a transparent sheath (Viewsite Brain Access Device, Vycor medical) as an access device to the hematoma, while hematoma is evacuated using a standard suction cannula and forceps if needed (22). The use of a transparent sheath carries the benefit of a 360° vision during hematoma evacuation which helps detect and evacuate residual hematoma around the sheath (31, 32). As observed in this pilot trial, future phase III trials should promote and adapt pragmatic, real-world eligibility criteria to facilitate enrolment and provide a real-world setting to enhance generalizability. Further patient and public involvement should be implemented to ensure adequate patient retention during the trial.

### 4.2 Safety and adverse events

We report three deaths (30%) and six adverse events (60%). The mortality rate is comparable to the current literature described for endoscopic hematoma evacuation ranging from 10 to 34.3% (16, 26, 31).

None of the adverse events were directly related to the surgery itself, but rather to the underlying disease. The most frequent adverse event was rebleeding, which occurred in two participants. Factors described to be potentially associated with rebleeding are timing of hematoma evacuation and the presence of a spot sign on the initial CT scan (38, 39). Controversies exist in the literature on what should be the ideal time of hematoma removal. Clearly, early evacuation should be the goal, since in hemorrhagic stroke reports show, that hematoma evacuation within 24 h leads to better functional outcome (18, 19). On the other hand, ultra early evacuation (<4 h) might lead to an unacceptable high percentage of rebleeding (40%) (40). Although some reports have shown that hematoma removal within 8 h might be safe, the rebleeding rate might still be higher than when surgery is delayed to 12 or even 24 h (40, 41). Furthermore, the current literature includes mostly people undergoing craniotomy and not endoscopic hematoma removal, making an inference to endoscopic surgery difficult (41). Based on the preliminary results of some endoscopic trials it seems that the rebleeding rate is overall lower than after surgical evacuation by craniotomy (26). However, earlier treatment could potentially reduce the secondary brain damage produced by toxic breakdown products and as such, the ideal time for ES, causing the least risk for rebleeding but the best chance for improving functional outcome, lies most likely somewhere within 24 h after bleeding onset, but overall still remains a matter of debate (7). We report a median time to surgery of 17 h, which is well within the 24-h time limit, however given that we did not include an active comparator or compared different time windows, no inference on whether 17 h are early enough or not can be concluded. Based on the time points of rebleeding and the eligibility criteria in this trial, it seems unlikely that the rebleeding was related to either early surgery or the presence of an arterial spot-sign. The ongoing studies on endoscopic evacuation of SSICH all have different time windows set for their cohorts, which will potentially provide us with more robust data on the ideal time point of surgical evacuation EVACUATE (NCT04434807), MIND (NCT03342664), and EMINENT-ICH (NCT05681988) (37, 42).

In the earliest endoscopic surgery reports, the presence of a spot sign was independently associated with intraoperative bleeding, requiring skill to manage the bleedings (38). Furthermore, in people with spot sing outcome at 90-days was poorer than in non-spot sign people, as it directly leads to rebleeding/hematoma enlargement (43, 44). However, recent publications have shown, that endoscopic surgery may mitigate the risk of intraoperative bleeding by direct cauterization and therefore surgery in people with a spot sign might not present a higher risk for rebleeding (26, 45).

### 4.3 Functional outcome

Although this pilot trial was not powered to show efficacy in improving functional outcome, we report comparable favorable outcome rates of ES (60%) with the current largest cohort of endoscopically operated participants in a single arm cohort (33). These observations are in line with previously reported data from several meta-analyses highlighting potential improvement of functional outcome rates in ES (16, 18, 46–49). Compared to other surgical procedures used in STICH I (mostly craniotomy) and MISTIE III (stereotactic catheter placement), ES in our cohort lead to higher rates of improved functional outcome (60% in ES vs. 44% in MISTIE III and 33% in STICH I) although based on our sample size this could be random at best (5, 12). The EMINENT-ICH trial is therefore designed to assess whether ES plus BMT compared to BMT alone improves functional outcome in people with SSICH.

### 4.4 Limitations

This study comes with several limitations inherent to the study design of any phase II feasibility study. First, we report no active control group, thus limiting the conclusion drawn from this study, besides conclusion on the surgical feasibility and safety. Furthermore, we used rather strict eligibility criteria, which restricted and slowed down our enrolment reduced the generalizability of our results. Due to the rather small sample size, drawing meaningful conclusions on the potential benefits of endoscopic hematoma evacuation on mortality and functional outcome is difficult.

Our pilot trial provides crucial data for the large multicenter randomized controlled trial EMINENT-ICH. The strengths of this phase II pilot trial are a meticulous screening log, screening every person that was admitted to our hospital with any kind of ICH. We used a standardized, published surgical protocol which made a uniform treatment approach possible. Lastly, we designed a pragmatic trial, adding only very little expenditure to the hospital staff, allowing adherence to the study procedures.

## 5 Conclusion

Early minimally invasive endoscopic surgery in intracerebral hemorrhage appears to be safe, feasible, and leads to satisfactory hematoma evacuation. Based on these results, EMINENT-ICH, a national, multicenter RCT has been launched, with the aim to assess the benefit of ES hematoma removal plus BMT on functional outcome and mortality compared to BMT alone.

## Data Availability

The raw data supporting the conclusions of this article will be made available by the authors upon reasonable request.
