# Supplementary material for: Early minimally invasive image-guided eNdoscopic evacuation of iNTracerebral hemorrhage: a phase II pilot trial
Source: Front Neurol. 2024 Nov 19;15:1484255. doi: 10.3389/fneur.2024.1484255 (PMC11611861; doi:10.3389/fneur.2024.1484255)
Supplement: Supplementary file 1 [file Data_Sheet_1.docx]

Supplementary Material

Early Minimally Invasive image-guided eNdoscopic Evacuation of iNTracerebral haemorrhage: A phase II pilot trial (EMINENT-ICH Pilot Trial)

Table of Contents

[1 Supplemental Table 1: Full eligibility criteria for the EMINENT-ICH Pilot Trial 3](#_Toc175127770)

[2 Supplemental Table 2: Visit schedule for the EMINENT-ICH Pilot Trial 5](#_Toc175127771)

[CONSORT 2010 checklist of information to include when reporting a pilot or feasibility trial* 6](#_Toc175127772)

# Supplemental Table 1: Full eligibility criteria for the EMINENT-ICH Pilot Trial

Inclusion criteria:

- Patient age ≥ 18 and <85
- No relevant disability prior to ICH (mRS 0-1 prior to ICH)
- Primary supratentorial deep or superficial intraparenchymal ICH of volume ≥ 20 mL < 100 mL (measured using the formula $\frac{A * B * C}{2}$) demonstrated on CT or MRI, with or without a component of intraventricular haemorrhage
- CT/MR demonstrates ICH stability (< 5 mL growth) at 6 hours after the admission scan if surgery is performed >6 hours after admission CT
- NIHSS ≥ 8 OR if a patient with a NIHSS<8 presents with at least one of the following deficits:
  - a severe hemiparesis (4 motor points on the NIHSS for facial palsy, motoric upper and lower extremities combined)
  - a severe motor or sensory aphasia (2 points on the NIHSS)
  - a profound hemi-inattention (formerly neglect, 2 points on the NIHSS)
  - a decreased level of consciousness (GCS<13)
- Presenting GCS 5 - 15
- Endoscopic haematoma evacuation can be initiated within 24 hours of symptom onset
- Systolic blood pressure can be controlled at <160 mmHg

Exclusion criteria:

- Imaging
  - "Spot sign" identified on CTA
  - Structural vascular or brain lesion as suspected cause of ICH, such as a vascular malformation (cavernous malformation, AVM etc), aneurysm, neoplasm
  - Haemorrhagic conversion of an underlying ischemic stroke
  - Infratentorial haemorrhage
  - Large associated intra-ventricular haemorrhage requiring treatment for related mass effect or shift due to trapped ventricle (extraventricular drainage [EVD] for intracranial pressure [ICP] management is allowed)
  - Midbrain extension/involvement
- Coagulation Issues
  - Oral or parenteral therapeutic anticoagulation which cannot be pharmacologically reverted until the planned time of evacuation
  - Known hereditary or acquired haemorrhagic diathesis, coagulation factor deficiency
  - Platelet count < 100 x 103 cells/mm3 or known platelet dysfunction
  - INR > 1.5 for any reason, elevated prothrombin time or activated partial thromboplastin time (aPTT), which cannot be corrected or otherwise accounted for (i.e., lupus anti-coagulant)
- Presenting GCS 3 or 4
- Requirement for emergent surgical decompression or uncontrolled ICP after EVD
- Unable to obtain consent from patient or appropriate surrogate (for patients without competence)
- Pregnancy, breast-feeding, or positive pregnancy test [either serum or urine] (Woman of child-bearing potential must have a negative history of current pregnancy prior to the study procedure)
- Evidence of active infection (indicated by fever ≥38°C) at the time of study inclusion
- Any comorbid disease or condition expected to compromise survival or ability to complete follow-up assessments through 180 days
- Based on physician’s judgment, patient does not have the necessary mental capacity to participate or is unwilling or unable to comply with protocol follow up appointment schedule
- Active drug or alcohol use or dependence

# Supplemental Table 2: Visit schedule for the EMINENT-ICH Pilot Trial

| Study period | Eligibility | Treatment, Intervention Period | | | | Follow-Up | |
| --- | --- | --- | --- | --- | --- | --- | --- |
| Visit | **0** | **1** | **2** | **3** | **4** | **5** | **6** |
| Time (hour, day, week) | **<18 hours** | **<24 hours** | **24 hours after treatment onset** | **72 hours after treatment onset** | **7 days after treatment onset** | **28-32 days after treatment onset** | **6 months after treatment onset** |
| Eligibility | X |  |  |  |  |  |  |
| Study consent | X |  |  |  |  |  |  |
| Vital signs  (BP,HR,height, Temp) | X | X | X | X | X | X | X |
| acquiring  NIHSS score | X | X | X | X | X | X | X |
| acquiring  GCS score | X | X | X | X | X | X | X |
| aquiring  mRS score | X | X | X | X | X | X | X |
| Blood samples  (NfL, GFAP, S100B, IL) |  | X | X | X | X | X | X |
| conducting  CT scan |  | X | X |  |  |  |  |
| Conducting MRI scan |  |  |  | X |  |  |  |
